# Prevalence and Predictors of Hepatic Steatosis in Patients Undergoing Sleeve Gastrectomy: A Biopsy-proven Study

**DOI:** 10.1007/s11695-025-08386-3

**Published:** 2025-11-28

**Authors:** Riham Soliman, Ahmed Helmy, Nabiel Mikhail, Helmy Ezzat, Ahmed Mehrez Gad, Ebrahim Abdel Halim, Khaled Zalata, Rokia Masoud, Ayman Hassan, Ahmed Farahat, Mohamed El Emam Abou Eisa, Mohamed Elbasiony, Gamal Shiha

**Affiliations:** 1 Tropical Medicine Dept., Faculty of Medicine, Port Said University, Port Said, Egypt; 2 Egyptian Liver Research Institute and Hospital (ELRIAH), Mansoura, Egypt; 3 Biostatistics and Cancer Epidemiology Dept.,South Egypt Cancer Institute , Assiut University, Assiut, Egypt; 4 Gastrointestinal Surgery Dept., Faculty of Medicine, Mansoura University, Mansoura, Egypt; 5 General Surgery Dept., Faculty of Medicine, Delta University for Science and Technology, Mansoura, Egypt; 6 General Surgery Dept., Faculty of Medicine, Horus University, New Damietta, Egypt; 7 Pathology Dept., Faculty of Medicine, Mansoura University, Mansoura, Egypt; 8 Higher Technological Institute of Applied Health Science, Sherbin, Egypt; 9 Gastroenterology and hepatology Unit, Internal Medicine Dept., Faculty of Medicine , Mansoura University, Mansoura, Egypt; 10 Tropical Medicine and Gastroenterology Dept., Faculty of Medicine, Assiut University, Assiut, Egypt

**Keywords:** Steatosis, Liver biopsy, CAP, Bariatric surgery

## Abstract

To date, there is a lack of population-based studies assessing the prevalence and severity of steatosis and metabolic-associated steatohepatitis (MASH) in Egypt. CAP is widely used as a non-invasive tool for hepatic steatosis assessment, yet its reliability in obese populations remains unclear. We aimed to examine the prevalence and risk factors for steatosis and steatohepatitis in Egyptian patients undergoing laparoscopic sleeve gastrectomy and evaluate CAP’s diagnostic accuracy against liver biopsy in detecting hepatic steatosis. Methods: In this prospective cross-sectional study (2019–2023), 162 obese adults undergoing bariatric surgery were enrolled. CAP was performed prior to intraoperative wedge liver biopsy. Histological grading of steatosis and NAS scoring were conducted by blinded pathologists. Diagnostic accuracy of CAP was evaluated using AUROC, sensitivity, specificity, PPV, and NPV. Results: Hepatic steatosis was present in 63.6% of patients by liver biopsy. CAP overestimated steatosis in 40% of biopsy-confirmed S0 cases, misclassifying them as S3. CAP cutoff of 286 dB/m for ≥ S1, sensitivity, specificity, PPV, and NPV were 57.7%, 65.0%, 76.3%, and 44.1%, respectively (AUROC = 0.577). Only 14.2% had steatohepatitis. Multivariate analysis identified albumin (*p* = 0.040) and hemoglobin (*p* = 0.018) as independent protective factors. Conclusion: CAP significantly overestimated steatosis severity highlighting its limited reliability in obese populations.

## Introduction

Obesity affects approximately 25% of the global population and over 50% of individuals with metabolic disorders, are increasingly recognized as a leading cause of chronic liver disease and subsequent complications [1]. A group of international experts suggested renaming fatty liver diseases to ‘Metabolic Dysfunction-Associated Fatty Liver Disease’ (MAFLD) to emphasize the complex interaction between fatty liver and metabolic dysfunctions. They have introduced new diagnostic criteria for MAFLD that require the detection of hepatic steatosis along with one or more of the following conditions: overweight/obesity, type 2 diabetes, or signs of metabolic dysregulation [2]. Recently, a multi-society Delphi consensus introduced the new nomenclature MASLD. This new nomenclature is considered less stigmatizing, more affirmative, and adequately reflects the role of metabolic dysfunction in the disease spectrum and is defined by evidence of hepatic steatosis and at least 1 of 5 cardiometabolic risk factors [3]. 

The clinical presentation of MALD includes simple steatosis and metabolic-associated steatohepatitis (MASH), which can evolve into fibrosis, cirrhosis, and hepatocellular carcinoma (HCC) [4]. Notably, HCC can develop even in patients who do not have cirrhosis [5]. Consequently, effective management of MASH is essential once it is identified. A weight reduction of over 10% has been demonstrated to be an effective treatment for MASH. Nevertheless, maintaining lifestyle modifications over a prolonged period is challenging, and the reoccurrence of obesity post-weight loss is common. Bariatric surgery presents a viable treatment alternative for MASH, offering a potential avenue to halt the progression towards terminal liver disease [6]. 

Non-invasive diagnosis of steatosis can be determined by the following: conventional B-mode ultrasonography [7], magnetic resonance imaging (MRI), magnetic resonance spectroscopy (MRS), Controlled attenuation parameter (CAP) [8], and biomarkers, e.g., the Fatty Liver Index (FLI) [9], Hepatic Steatosis Index (HSI) [10]. Traditionally, liver biopsy has served as the gold standard for identifying and assessing the severity of liver steatosis [11]. 

Laparoscopic Sleeve gastrectomy, as a bariatric surgery procedure, induces long-term excess weight loss of up to 30% and remission of diabetes mellitus while reducing cardiovascular and cancer-related mortality, the two most frequent causes of death in patients with steatohepatitis [12]. Studies reported the presence of steatosis and steatohepatitis in obese adults before weight loss surgery about 80.2% to 90% [13]. 

Although obesity and metabolic disorders are prevalent in Egypt, the epidemiological burden of hepatic steatosis and steatohepatitis has not been systematically studied in the Egyptian population. To date, there is a lack of large-scale population-based studies assessing the prevalence and severity of steatosis and metabolic-associated steatohepatitis (MASH) in Egypt. This underscores the importance of evaluating liver disease burden in high-risk populations, such as patients undergoing bariatric surgery, to better understand the local disease landscape and inform evidence-based public health planning and clinical management protocols.

Our primary objective was to study the prevalence and a risk factors of hepatic steatosis and steatohepatitis in obese patients undergoing sleeve gastrectomy.Secondary objective is to assess the diagnostic performance of the controlled attenuation parameter (CAP) in detecting hepatic steatosis compared to liver biopsy in this cohort.

## Patients and Methods

This is a prospective, cross-sectional, single-center study conducted between May 2019 and December 2023. It involved consecutive patients who underwent laparoscopic sleeve gastrectomy at the Egyptian Liver Research Institute and Hospital (ELRIAH) in Sherbin, Mansoura, Egypt. This study followed the Declaration of Helsinki, its 2008 revisions, and the International Conference on Harmonization recommendations on Good Clinical Practice.

### Inclusion and Exclusion Criteria

Inclusion criteria were as follows: patients undergoing laparoscopic sleeve gastrectomy ≥ 18 years of age, able to give written informed consent, and were scheduled to have a liver biopsy. BMI ≥ 40 or BMI ≥ 35 with at least one obesity-related comorbid conditions (including type-2 diabetes mellitus (DM), hypertension (HTN), hyperlipidemia, obstructive sleep apnea (OSA), GERD, asthma, Chronic venous insufficiency, severe osteoarthritis or considerably impaired quality of life) [14]. Recent updates have included patients with a BMI of 30–35 with uncontrollable type 2 diabetes or metabolic syndrome as an indication for a laparoscopic sleeve gastrectomy [15]. Medical weight loss treatment was given prior to bariatric surgery.

**Fig. 1:**
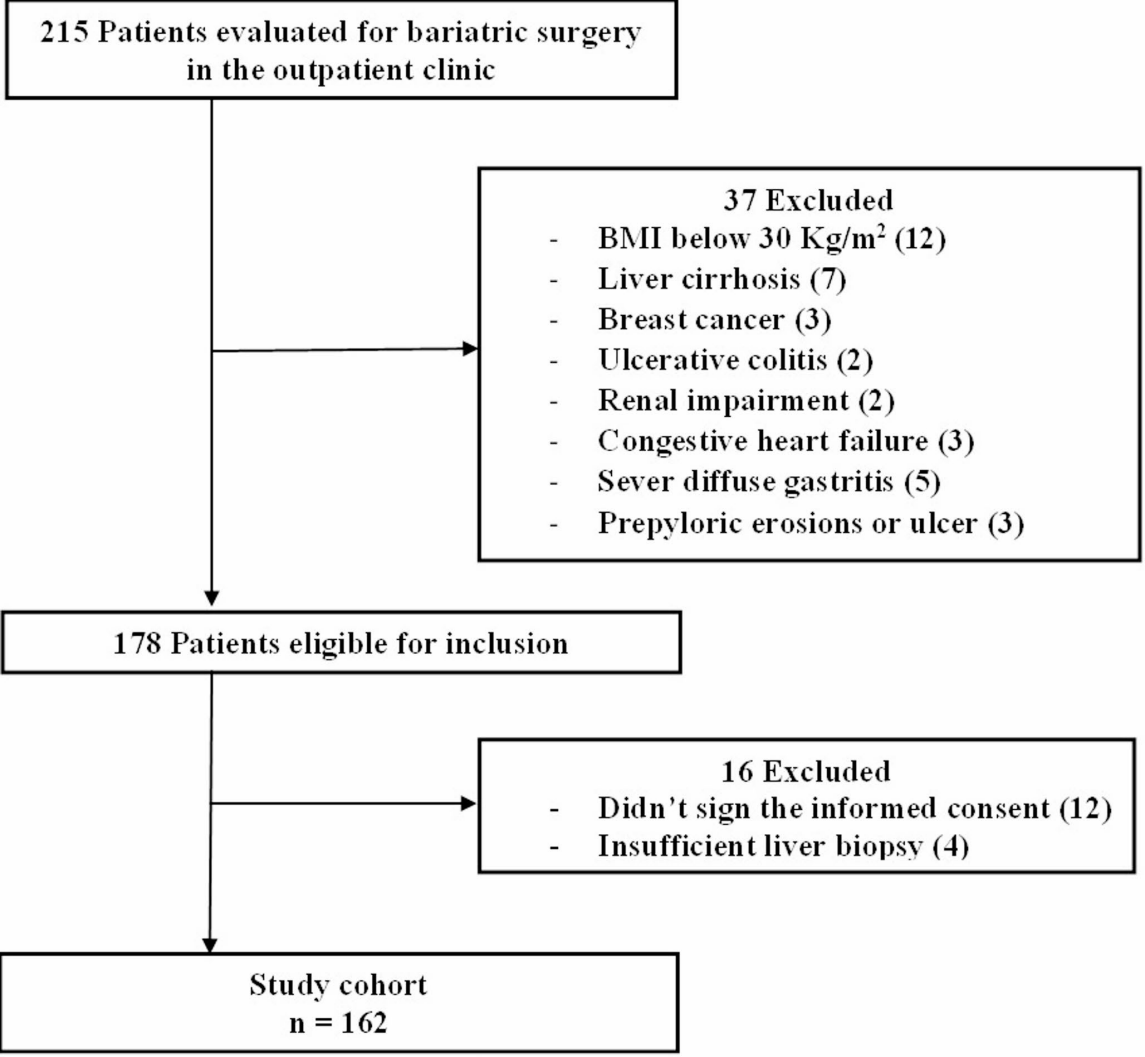
Flowchart of patients included in the study

Individuals were excluded from the study if they had history of liver transplantation, congestive heart failure or significant valvular heart disease, ongoing alcohol consumption, active malignancy or other terminal illnesses, and refusal to provide informed consent or undergo venipuncture. Patients with coexisting hepatic disorders of autoimmune, cholestatic, or viral etiology, as well as those previously treated with systemic anti-obesity pharmacotherapy, were also excluded. Patients flow chart (Fig. 1).

## FibroScan LSM and CAP

FibroScan examination was performed by physicians trained and certified by the manufacturer. The Fibro Scan Fibro Scan 502 (Echosens, Paris, France). device simultaneously measures LSM and CAP using VCTE technology. Transient elastography results were considered reliable when the following criteria were met: (a) 10 successful measurements; (b) an interquartile range (IQR) lower than 30% of the median value; and (c) a success rate of > 60% [16]. For high BMI (≥ 30 kg/m2), examination with the XL probe, with two experienced operators, was done [17]. Transient elastography was done by FibroScan 502 (Echosens, Paris, France). FibroScan was performed within 2–4 weeks before obtaining the laparoscopic liver biopsy (LB) during the sleeve gastrectomy procedure.

## Liver Biopsy

A simple technique for wedge biopsy of the liver, which can be performed concomitantly with laparoscopic surgery. For this technique, the edge of the liver is clamped with two standard laparoscopic bowel graspers at an approximately 90-degree angle to create a wedge of hepatic tissue for biopsy - the biopsy size is at least 2 cm. Endoscopic scissors are used to excise the specimen between the graspers. The cutting surface of the liver were coagulated with a monopolar device. This technique is rapid, safe, and inexpensive and requires no specific instruments or devices.

## Histopathologic Evaluation

LB slides were analyzed independently by two experienced pathologists blinded to each other’s reading and the patient’s clinical and Fibro Scan data. They reviewed the slides together to reach a consensus in case of disagreement. Each LB specimen was considered adequate if incorporates at least 20 mm core tissue or 11 portal tracts [18]. Fibrosis was staged on a 0–4 scale (METAVIR): F0, no fibrosis; F1, portal fibrosis without septa; F2, portal fibrosis and few septa; F3, numerous septa without cirrhosis; and F4, cirrhosis [19]. Steatosis was scored on a 0–3 scale: grade S0, < 5%; grade S1, 5%- 33%; grade S2, > 33%−66%; and grade S3, > 66% [18]. NAS score was calculated by the sum of scores of steatoses (0–3), lobular inflammation (0–3) and hepatocyte ballooning (0–2) [20]. Patients were classified as having no NASH (NAS score 0–3), borderline NASH (NAS score 4) and NASH (NAS score 5–8).

### Statistical Analysis

For descriptive statistics, continuous variables were expressed as median (interquartile range (IQR) and categorical variables as absolute figures with percentages. Cox-regression analysis was used for univariate and multivariate analyses. Confidence intervals (CIs) were reported at the 95% level. Diagnostic accuracy indices and area under the receiver operator characteristics (AUROC) curve for CAP was also measures. P values of < 0.05 were considered statistically significant. Only patients with histology data were analyzed. Additionally, no attempt has been made to replace any missing data. All analyses were performed using the Statistical Package for Social Sciences (SPSS) *version* 26, IBM Corp., USA.

## Results

A total of 215 patients were initially evaluated for eligibility. After applying inclusion and exclusion criteria, 162 consecutive patients with complete liver biopsy data were enrolled in the study. All patients underwent elective laparoscopic sleeve gastrectomy between May 2019 and December 2023. Among the included cohort, 48 patients (29.6%) were male, the median age was 35 years, and the median BMI was 39.1 kg/m². Notably, 9.9% of patients had a diagnosis of type 2 diabetes mellitus. Detailed baseline demographic and clinical characteristics are presented in Table 1.

**Table 1:** All patients’ characteristics and based on the presence or absence of steatosis

Variable	Unit/Category	All *n* = 162 (22.0)	With steatosis *n* = 103(63.6)	Without steatosis *n* = 59 (36.4)	*P* value Univariate	*P* value Multivariate	AUROC (95%%CI)
Age	Years	35 (29–43)	36 (32–43)	31 (24–43)	**0.041**	0.949	
Gender	Male Female	48 (29.6) 114 (70.4)	38 (36.9) 65 (63.1)	10 (16.9) 49 (83.1)	**0.007**	0.586	
Diabetes Mellitus	Yes No	146 (90.1) 16 (9.9)	89 (86.4) 14 (13.6)	57 (96.6) 2 (3.4)	**0.036**	0.988	
Body Mass Index	kg/m^2^	39.1 (33.1–47.7)	41.1 (32.3–47.8)	38.1 (33.8–47.4)	0.800		
ALT	IU/L	16.5 (12.0–25.1.0.1)	17.5 (12.2–29.8)	16.1 (11.3–23.0)	0.217		
AST	IU/L	17.0 (13.4–22.0)	17.0 (14.0–23.5.0.5)	16.8 (13.0–21.0)	0.215		
ALP	IU/L	73.0 (57.0–92.0)	73.0 (56.0–89.9.0.9)	77.5 (62.8–97.7)	0.369		
Total Bilirubin	mg/dL	0.53 (0.40–0.82)	0.57 (0.43–0.80)	0.53 (0.40–0.82)	0.968		
Albumin	g/dL	4.3 (4.1–4.5)	4.4 (4.1–4.6)	4.2 (4.0–4.4.0.4)	**0.008**	**0.040**	0.635 (0.539–0.730)
Platelets count	x10^3^/mL	251 (204–309)	247 (204–310)	254 (203–299)	0.789		
Hb	g/dL	12.4 (11.8–13.5)	12.7 (11.9–13.7)	12.2 (11.5–12.9)	**0.008**	**0.018**	0.626 (0.539–0.713)
RBCs	x10 ^6^/mL	4.7 (4.4–5.0.4.0)	4.8 (4.5–5.1)	4.6 (4.3–5.0.3.0)	0.027	0.461	
WBCs	x10 ^3^/mL	7.4 (5.5–10.8)	7.7 (5.8–11.3)	7.2 (5.3–10.5)	0.500		
LSM	kPa	5.3 (4.2–7.4)	4.7 (3.8–5.8)	5.3 (4.2–7.4)	0.470		
CAP	dB/m	284.5 (206.8–340.0)	298.5 (210.0–342.5.0.5)	252.0 (192.5–331.5.5.5)	0.173		
Cholesterol	mg/dL	195 (157–236)	197 (174–242)	184 (148–224)	0.053		
Triglycerides	mg/dL	149 (104–211)	147 (115–204.5.5)	152 (92–214)	0.799		
HDL	mg/dL	35 (26–45)	35 (26–47)	35 (25.8–42)	0.361		
LDL	mg/dL	124 (92–154)	127 (94–167)	114 (84–145)	0.095		
HOMA-IR index		2.2 (1.3–4.2)	2.45 (1.60–4.75)	1.65 (1.23–3.60)	**0.038**	0.990	
NAS score	0 1 2 3 4 5 6 7 8 8	96 (59.3%) 8 (4.9%) 12 (7.4%) 23 (14.2%) 15 (9.3%) 5 (3.1%) 3 (1.9%) 0 (0.0%) 0 (0.0%)	37 (35.9%) 8 (7.8%) 12 (11.7%) 23 (22.3%) 15 (14.6%) 5 (4.9%) 3 (2.9%) 0 (0.0%) 0 (0.0%)	59 (100.0%) 0 (0.0%) 0 (0.0%) 0 (0.0%) 0 (0.0%) 0 (0.0%) 0 (0.0%) 0 (0.0%) 0 (0.0%)	**< 0.001**	**< 0.001**	0.806 (0.738–0.874)

*Distribution is expressed as median (interquartile range; IQR) or number (percentage). ALP; alkaline phosphatase*, ALT: alanine aminotransferase. AST: aspartate aminotransferase. CAP: controlled attenuation parameter. Hb; hemoglobin. HDL & LDL: High-and low-density lipoprotein. LSM; liver stiffness measure. NAS: nonalcoholic steatohepatitis scoring. *; by METAVIR Score.

Comparison between patients with and without steatosis regarding demographic, clinical, laboratory, histopathological, LSM, CAP and NAS score characteristics in shown in Table 1. According to univariate regression analysis, steatosis was associated with older (36.0 vs. 31.0 years, *p* = 0.041), being male (36.9% vs. 16.9%, *p* = 0.007), presence of DM (13.6% vs. 3.4% *p* = 0036), higher HOMA-IR index (2.45 vs. 1.65, *p* = 0.038). higher hemoglobin levels (12.7 vs. 12.2 g/dL, *p* = 0.008), higher serum albumin levels (4.4 vs. 4.2 g/dL, *p* = 0.008), and higher RBCs counts (4.8 vs. 4.6 × 10^6^/mL). However, on multivariate regression analysis, only albumin and hemoglobin were found to be significant; *p* = 0.040 and *p* = 0.018 respectively, with AUROC curve (95%CI) of 0.635 (0.539–0.730) and 0.626 (0.539–0.713) respectively, Table 1.

Regarding NASH, 139 patients (85.8%) had no NASH (NAS score of 0–3), 15 patients (9.3%) had borderline NASH, and only eight patients (4.9%) had definite NASH (Table 2). Univariate comparison of patient characteristics according to presence of NASH showed only significant differences as regards gender (25.5% males with no NASH vs. 50.0% males with NASH, *p* = 0.008), cholesterol level (194 mg/dL in those with no NASH compared to 253 mg/dL for those with NASH, *p* = 0.010) and median (IQR) CAP being 266 (199–324) and 327 (298–337) in patients without NASH vs. those with NASH respectively (*p* = 0.049).

**Table 2:** Patients’ characteristics based on the presence or absence of steatohepatitis

Variable Variable	Unit or Category	No NASH NAS score = 0–3 *n* = 139 (85.8)	Borderline NAS score = 4 *n* = 15 (9.3)	NASH NAS score = 5–8 *n* = 8 (4.9)	*P* value Univariate
Age		35 (29–43)	34 (25–45)	39.5 (33.3–43.0)	0.684
Gender	Male Female	35 (25.2) 104 (74.8)	9 (60.0) 6 (40.0)	4 (50.0) 4 (50.0)	**0.008**
Diabetes Mellitus	No Yes	126 (90.6) 13 (9.4)	12 (80.0) 3 (20.0)	8 (100.0) 0 (0.0)	0.266
Body Mass Index	kg/m^2^	38.7 (32.5–46.6)	46.0 (35.3–50.1)	45.8 (33.9–55.2)	0.404
ALT	IU/L	16 (12–25)	32 (15–48)	15 (9–33)	0.136
AST	IU/L	17 (13–21)	26 (15–30)	16 (14–29)	0.213
ALP (IU/L)	IU/L	73 (56–93)	75.0 (51–101)	79 (71–88)	0.871
Total Bilirubin	mg/dL	0.50 (0.40–0.82)	0.70 (0.47–1.10)	0.40 (0.30–0.60)	0.089
Albumin	g/dL	4.3 (4.1–4.5)	4.2 (4.1–4.5)	4.5 (4.2–4.6)	0.544
Cholesterol	mg/dL	194 (155–231)	191 (131–207)	253 (227–507)	**0.010**
Triglycerides	mg/dL	147 (102.5–212.5.5.5)	140 (118–184)	207.5 (170.5–326.8.5.8)	0.051
HDL	mg/dL	35 (26–45)	34 (15–42)	38 (27–54)	0.495
LDL	mg/dL	123 (92–153)	121 (86–149)	170 (92–190)	0.398
HOMA-IR index		2.20 (1.30–3.95)	3.45 (1.56–5.90)	1.80 (1.30–2.7)	0.430
Platelets count	x10^3^/mL ^3^/mL	247 (201–305)	266 (225–319)	313 (246–359)	0.180
Hb	g/dL	12.3 (11.7–13.3)	13.5 (12.3–13.8)	12.7 (11.8–13.8)	0.078
RBCs	x10 ^6^/mL	4.7 (4.4–5.0.4.0)	4.9 (4.6–5.5)	4.9 (4.6–5.5)	0.259
WBCs	x10 ^3^/mL	7.3 (5.5–10.3)	8.8 (6.0–13.9.0.9)	8.2 (7.1–15.1)	0.363
CAP	dB/m	266 (199–324)	357 (228–389)	327 (298–337)	**0.049**

*Distribution is expressed as median (interquartile range; IQR) or number (percentage). ALP; alkaline phosphatase*, ALT: alanine aminotransferase. AST: aspartate aminotransferase. CAP: controlled attenuation parameter. Hb; hemoglobin. HDL: high-density lipoprotein. LDL: low-density lipoprotein. LSM; liver stiffness measure. NAS: nonalcoholic steatohepatitis scoring. NASH: nonalcoholic steatohepatitis. RBCs; red blood cells. WBCs: white blood cells. *; by METAVIR Score.

**Fig. 2:**
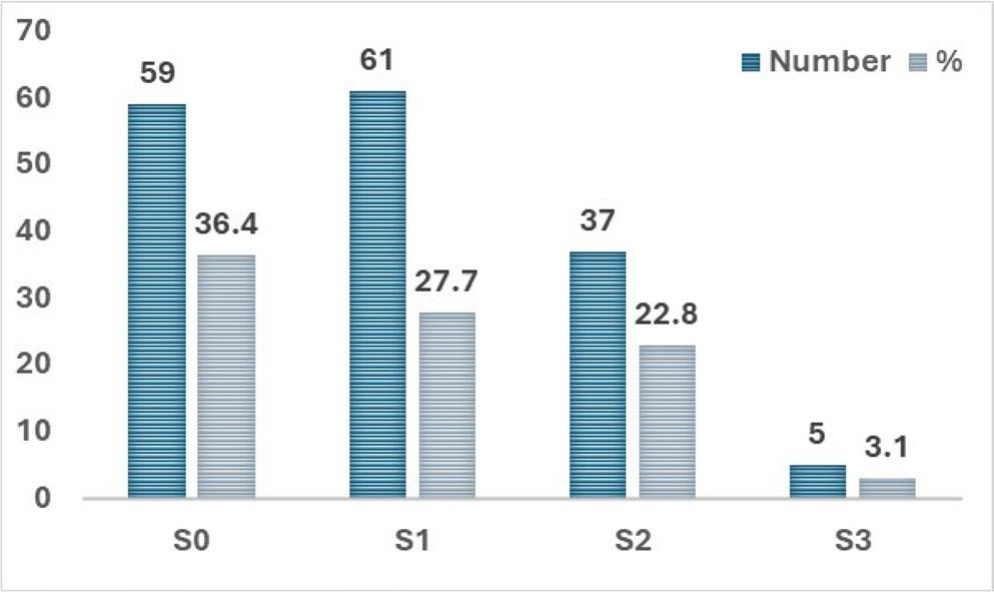
Frequency distribution of hepatic steatosis grades as assessed by liver biopsy in the studied cohort (*n* = 162)

Among the 162 patients who underwent liver biopsy during laparoscopic sleeve gastrectomy, hepatic steatosis was identified in 103 individuals (63.6%). The distribution of steatosis grades was as follows: 37.7% had mild steatosis (S1), 22.8% moderate (S2), and 3.1% severe (S3), while 36.4% of patients exhibited no histological steatosis (S0) as shown in Fig. 2. Additionally, only 14.2% of the patients with steatosis were found to have steatohepatitis. Absence of steatosis was significantly associated with younger age (*p* = 0.041), female sex (*p* = 0.007), lower prevalence of type 2 diabetes mellitus (*p* = 0.036), lower HOMA-IR index (*p* = 0.038), and lower serum albumin (*p* = 0.008) and hemoglobin levels (*p* = 0.008). Multivariate analysis confirmed serum albumin (*p* = 0.040) and hemoglobin (*p* = 0.018) as independent protective factors. Notably, body mass index (BMI), liver enzymes (ALT, AST), and lipid profile parameters did not differ significantly between patients with and without steatosis.

**Fig. 3:**
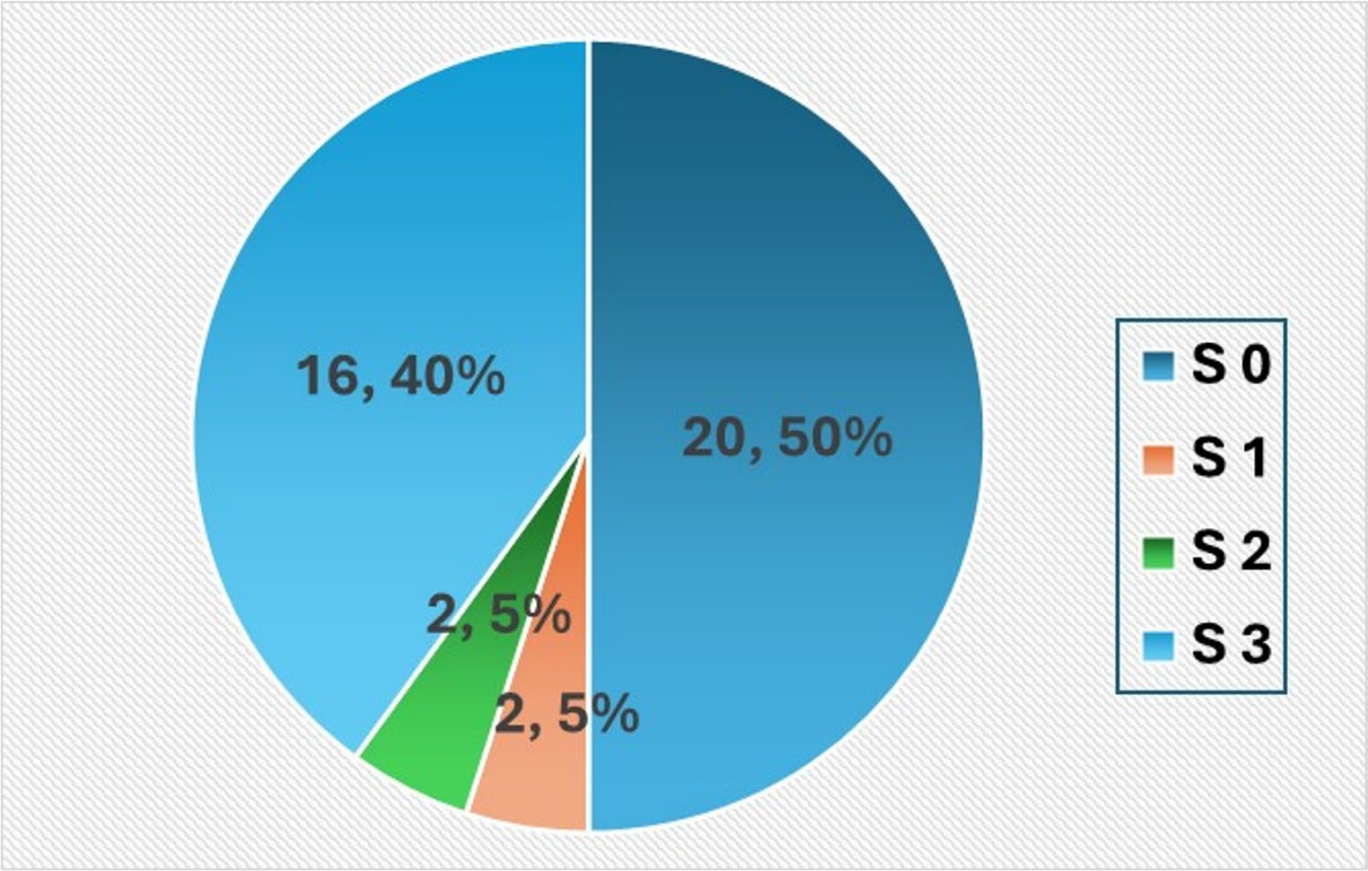
Frequency distribution of the CAP results in patients with no steatosis by liver biopsy (S = 0)

Among the 59 patients (36.4%) who had no steatosis (S0) on liver biopsy, CAP results by Fibro Scan were available for 40 individuals. Of these, only 50% were correctly identified as S0 by CAP. Notably, 40% were misclassified as having severe steatosis (S3), while 5% were categorized as S1 and another 5% as S2. (Fig. 3)

**Fig. 4:**
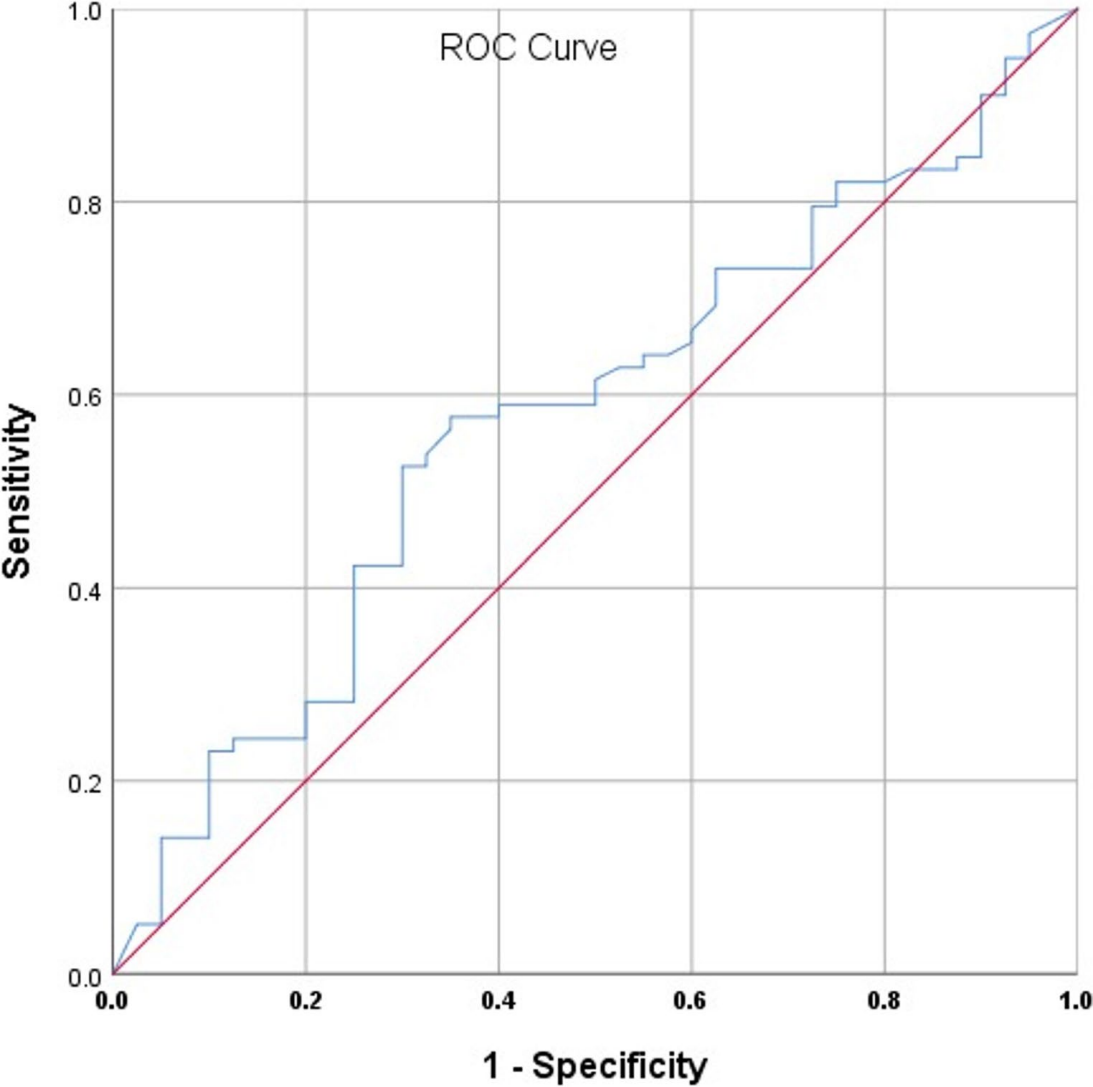
Area under the receiver operator characteristic (AUROC) curve (95% CI) for CAP and steatosis ≥ 1 (≥ 5%), 0.577 (0.469–0.685)

As shown in Tables 3 and 4, the diagnostic accuracy indices; sensitivity, Specificity, PPV and NPV of CAP at a cutoff value of 286 dB/m in the detection of steatosis ≥ S1 are 0.577 (0.460–0.688), 0.650 (0.483–0.794), 0.763 (0.618–0.839) and 0.441 (0.330–0.620) respectively with an area AURC curve of 0.577 (95%CI: 0.469–0.685, Fig. 4).

**Table 3:** CAP results in patients without steatosis (S0) by liver biopsy

	Controlled Attenuation Parameter (CAP)		
Steatosis Grade by liver biopsy	Patients’ number	Percent*	Valid percent**
S 0	20	33.9	50.0
S 1	2	3.4	5.0
S 2	2	3.4	5.0
S 3	16	27.1	40.0
Total	40	67.8	100.0
Missing CAP results	19	32.2	
Total	59	100.0	

*; out of 40. **; out of 59.

**Table 4:** Diagnostic performance of CAP for steatosis grades ≥ 1

Steatosis grade (S) ≥ S1 by Liver Biopsy	
AUROC Curve (95% CI)	0.577 (0.469–0.685)
Cutoff value (dB/m) Sensitivity (95% CI) Specificity (95% CI) PPV (95% CI) NPV (95% CI)	286 0.577 (0.460–0.688) 0.650 (0.483–0.794) 0.763 (0.618–0.839) 0.441 (0.330–0.620)

*AUROC; area under the receiver characteristic curve. CI; confidence interval. NPV; negative predictive value PPV; positive predictive value.*

## Discussion

Our study showed significant diagnostic discrepancies between controlled attenuation parameter (CAP) and liver biopsy in assessing hepatic steatosis among bariatric surgery patients. While liver biopsy remains the gold standard, CAP demonstrated a tendency to over diagnose severe steatosis, with 40% of biopsy-confirmed S0 patients misclassified as S3. These findings emphasize the need for caution when using CAP as a standalone diagnostic tool for hepatic steatosis, particularly in populations with obesity, where its reliability may be affected by multiple factors.

The overestimation of severe steatosis by CAP observed in our cohort is consistent with prior studies that have raised concerns about its reliability in obese individuals [21–23]. A systematic review and meta-analysis of 61 studies involving 10,537 patients demonstrated that CAP’s diagnostic accuracy declines with increasing BMI, underscoring the limitations of this tool in this population [21]. These findings, along with ours, emphasize the importance of cautious interpretation of CAP results in obese patients and support the need for BMI-adjusted thresholds or alternative methods to enhance diagnostic precision.

CAP’s performance can be further influenced by technical factors such as subcutaneous fat thickness and variations in liver echogenicity due to fibrosis, both of which are prevalent in individuals with obesity. Studies have shown that subcutaneous fat thickness can attenuate ultrasound signals, leading to skewed CAP results [24]. Similarly, liver echogenicity changes due to fibrosis have been identified as confounding variables that impact CAP measurements [25]. These limitations underscore the importance of refining CAP diagnostic thresholds and integrating complementary diagnostic tools to improve accuracy in obese populations.

In line with our findings, the multicenter UK study by Eddowes et al. [26] demonstrated that while CAP has good diagnostic accuracy for detecting the presence of hepatic steatosis (S ≥ 1), its performance in differentiating higher steatosis grades—particularly between S2 and S3—is limited. Their data showed considerable overlap in CAP values between moderate and severe steatosis, with a minimal difference in optimal cutoff thresholds (331 vs. 337 dB/m) and a lack of statistical significance between the two grades.

The protective role of younger age and female sex in the absence of hepatic steatosis is consistent with established evidence of estrogen’s hepatoprotective effects against fat accumulation through its regulatory effects on lipid metabolism and adipocyte function, a benefit that diminishes after menopause [27]. This hormonal influence contributes to the lower prevalence of NAFLD in premenopausal women compared to men or postmenopausal women.

Additionally, the absence of a significant association between BMI and steatosis in our study supports the notion that visceral adiposity, rather than overall adiposity, plays a critical role in hepatic steatosis development. Prior research highlights visceral fat as a key driver of metabolic dysfunction and ectopic fat deposition, reinforcing its significance in the pathogenesis of NAFLD [28]. 

Also, younger age and lower HOMA-IR index are well-known protective factors against steatosis because they typically reflect more favorable metabolic health, and are characterized by more efficient fat oxidation, reduced lipogenesis, and less systemic inflammation. Indeed, insulin sensitivity (lower HOMA-IR) reduces the activation of pathways leading to fat accumulation in the liver, such as those triggered by hyperinsulinemia [29]. It has also been reported in a systematic review of 798 patients that young adults are more prone to store fat in subcutaneous tissue and reach the threshold of bariatric surgery indication before their liver is damaged [30]. Our results showed that NASH was significantly linked to the level of cholesterol (0.010) which was in agreement with Walenbergh and Shiri-Sverdlov (2015), as they found that free cholesterol is more linked to steatohepatitis than free fatty acids or TGs, and they proposed it as a risk factor for steatohepatitis [31]. 

In our study, 103 patients (63.6%) had no NASH (NAS score of 0–3) and 23 patients (14.2%) had borderline or clear NASH (NAS score of 4–8). This contrasts with Souto et al. (2018) who reported NASH in 47.2% of their patients.^46^ A meta-analysis conducted by Barbois et al. (2017) showed that the prevalence of NASH in bariatric surgery patients is 25%, and high ALT and AST are independent risk factors [32]. This may explain the low prevalence of NASH in our study as our median ALT and AST levels were 16.5 IU/L and 17 IU/L respectively.

Our results also showed that albumin, and hemoglobin, are significantly associated with steatosis (*p* = 0.040 and *p* = 0.014 respectively), these finding in agreement with Abdollahi et al. (2023) that concluded that higher albumin in mice model may be linked to higher steatosis risk, as albumin carries free fatty acids and increases their delivery to the liver [33], and Juárez-Hernández et al. (2018) who reported higher hemoglobin has been linked to steatosis in Mexican subjects, which may be a counter-acting mechanism to protect against the harmful effect of steatosis, as hemoglobin has shown antioxidant protective effect [34]. 

This study’s strengths include its prospective design and the use of liver biopsy as the diagnostic gold standard. The short interval between CAP measurements and biopsy (14 days) minimizes temporal variability, enhancing the reliability of our findings. Furthermore, rigorous selection criteria eliminated confounding factors such as alcohol consumption, hepatic overlapping syndromes with other liver diseases, and elevated liver enzyme levels at the time of evaluation. These measures distinguish our study from others conducted in Western populations, where alcohol intake and overlapping conditions often complicate the assessment of hepatic steatosis. Lastly, the use of wedge biopsies provided sufficient liver tissue, thereby reducing the risk of sampling error and enhancing the reliability of histopathological assessment.

Our study has limitations; a single-center investigation, the generalizability of findings to broader populations may be limited.

Future research should focus on refining CAP diagnostic thresholds for obese populations and investigating alternative non-invasive methods that account for subcutaneous fat thickness and fibrosis-related echogenicity. Multicenter studies involving diverse populations are essential to validate these findings and improve the generalizability of results. Additionally, integrating CAP with other diagnostic modalities, such as serum biomarkers or advanced imaging techniques, may enhance its clinical utility. Longitudinal studies could further clarify the metabolic and hormonal factors, such as estrogen decline in postmenopausal women, that influence hepatic fat accumulation and its detection.

In conclusion, this study demonstrates significant diagnostic discrepancies between CAP and liver biopsy in assessing hepatic steatosis, with CAP frequently over-diagnosing severe steatosis. By addressing CAP’s limitations, including the influence of technical factors like subcutaneous fat and fibrosis, and revisiting its diagnostic thresholds, we can enhance its reliability and clinical applicability. These efforts are crucial for improving non-invasive diagnostic tools and optimizing management strategies in obese populations. The high prevalence of steatosis (63.6%) in our cohort and the absence of associations with BMI highlight the importance of visceral adiposity and hormonal factors in hepatic fat deposition. The prevalence of steatosis in obese patients who underwent laparoscopic sleeve gastrectomy was only 63%; with only 14.2% had NASH. This low prevalence of NASH and steatohepatitis could be linked to the female sex predominance, younger age, low prevalence of DM, lower HOMA-IR, and higher albumin and hemoglobin levels.

## Data Availability

The datasets used and/or analysed during the current study are available from the corresponding author on reasonable request.

## Abbreviations

ALP: alkaline phosphatase
ALT: alanine aminotransferase
AST: aspartate aminotransferase
CAP: Controlled Attenuation Parameter
DM: diabetes mellitus
Hb: hemoglobin
HDL: high-density lipoprotein
IQR: interquartile range
LDL: low-density lipoprotein
LSM: liver stiffness measure
MASLD: Metabolic Associated Steatosis and Liver Disease
NAS: nonalcoholic steatohepatitis scoring
NASH: nonalcoholic steatohepatitis
SPSS: statistical package for social sciences
WBCs: white blood cells
